# Effect of Ambient Temperature on Australian Northern Territory Public Hospital Admissions for Cardiovascular Disease among Indigenous and Non-Indigenous Populations 

**DOI:** 10.3390/ijerph110201942

**Published:** 2014-02-13

**Authors:** Leanne Webb, Hilary Bambrick, Peter Tait, Donna Green, Lisa Alexander

**Affiliations:** 1 Climate Change Research Centre, University of New South Wales, Sydney, NSW 2052, Australia; E-Mails: leanne.webb@csiro.au (L.W.); l.alexander@unsw.edu.au (L.A.); 2 Centre for Health Research, School of Medicine, University of Western Sydney, Sydney, NSW 2560, Australia; E-Mail: h.bambrick@uws.edu.au; 3 Public Health Association of Australia, Deakin, ACT 2600, Australia; E-Mail: aspetert@bigpond.com; 4 ARC Centre of Excellence for Climate System Science, University of New South Wales, Sydney, NSW 2052, Australia

**Keywords:** Indigenous health, hospital admissions, climate, ambient temperature, cardiovascular disease, Australia

## Abstract

Hospitalisations are associated with ambient temperature, but little is known about responses in population sub-groups. In this study, heat responses for Indigenous and non-Indigenous people in two age groups were examined for two categories of cardiac diseases using daily hospital admissions from five Northern Territory hospitals (1992–2011). Admission rates during the hottest five per cent of days and the coolest five per cent of days were compared with rates at other times. Among 25–64 year olds, the Indigenous female population was more adversely affected by very hot days than the non-Indigenous female population, with admission rates for ischaemic heart disease (IHD) increasing by 32%. People older than 65 were more sensitive to cold, with non-Indigenous male admissions for heart failure increasing by 64%, and for IHD by 29%. For older Indigenous males, IHD admissions increased by 52% during cold conditions. For older non-Indigenous females, increases in admissions for heart failure were around 50% on these cold days, and 64% for older Indigenous females. We conclude that under projected climate change conditions, admissions for IHD amongst younger Indigenous people would increase in hot conditions, while admissions among elderly people during cold weather may be reduced. The responses to temperature, while showing significant relationships across the Northern Territory, may vary by region. These variations were not explored in this assessment.

## 1. Introduction

Human physiology is directly affected by variability in temperature [1,2,3], with extremes having the potential to cause significant heat and cold stress related illness and increased mortality [4,5]. For example, in the 2003 summer European heatwave, between 22,000 to 45,000 additional heat related deaths were reported to have occurred [6,7]. During the more recent Russian summer heatwave in 2010, a death toll of 55,000 was estimated [8]. Unsurprisingly, concerns have been raised about greater impacts on human health as climate change projections suggest more frequent and/or more intense heatwaves will occur during the summer months [9]. 

In Australia, studies of the effects of extreme heat on population health reviewed by Bi *et al.* [10] found that although Australians are somewhat acclimatised to hot summers and regular heatwaves, morbidity and mortality associated with extreme heat remains a regular occurrence [11]. The major impacts from extreme heat have been documented to impact the population in a number of ways including increased admission rates for renal disease [12,13], cardiovascular disease [14,15,16,17,18], and mental health [18,19].

Extremes in cold conditions also affect human health [20]. A study of Sicilian hospital admissions between 1987 and 1998 found a significant increase in admissions for acute myocardial infarction during winter [21]. Similarly an increase in heart failure admissions was reported over the winter in Scotland [22]. An increase in admissions for cardiovascular disease in people over the age of 65 following cold conditions was also reported in Canada [23]. 

The prevalence of long-term adverse health conditions is much higher among Indigenous than non-Indigenous people in Australia, with cardiovascular disease being the largest contributor to the disparity in health [24]. For example, ischaemic heart disease (IHD), consequent to coronary artery blockage, is 2.1 times more prevalent in Indigenous Australians. Heart failure is 1.7 times more prevalent in Indigenous than non-Indigenous Australians [24]. Both of these conditions are associated with lifestyle factors such as smoking and being overweight [25]. These risk factors are more prevalent in Indigenous populations [24].

It is anticipated that climate change will exacerbate current health disparities [26,27], especially since underlying chronic diseases such as IHD and heart failure are a contributing factor to higher morbidity and mortality from extremes in ambient temperature. Climate change projections indicate significant continent-wide increases in the frequency of heatwaves in Australia [28], with the largest increases occurring in inland and northern areas [29]. If climate change continues as projected on its current path [30] the number of days over 35 °C in Alice Springs, where there is a large Indigenous population, may be up to twice the current 90 days per year by 2070 [29]. Some potential health benefits may also arise in future climates due to fewer very cold days [20]. On balance, though, it is expected that adverse health impacts from climate warming are likely to dominate later into the century [31].

Until now, the potential impact of more extreme ambient temperature on Indigenous *vs.* non-Indigenous health has only had very limited quantification, though this population sub-group is potentially highly vulnerable to environmental extremes [32]. This is because there is a higher prevalence of underlying chronic health problems, over-crowding and limited access to safe water supplies found in this sub-population [33,34,35]. For some Indigenous communities, the remoteness and limited access to health services can also increase vulnerability [36]. In addition, socioeconomic disadvantage tends to reduce capacity to adapt to anthropogenic climate change. Specific qualitative concerns regarding these indirect impacts of climate change on Indigenous health have been reported in New Zealand [37], Canada [38] and Australia [26]. 

Understanding the connection between observed temperatures and hospital admissions may be helpful to assess the likely impacts of future climate change on the health of specific sub-groups, and therefore would enable improved focusing of health care services and policy action. Quantifying these associations could improve health promotion, prevention and care delivery services, especially in regional and remote areas. 

If the health of Indigenous Australians in the Northern Territory is more sensitive to more extreme ambient temperature conditions than the non-Indigenous population, health disparities will be exacerbated by climate change. To explore this supposition, this study focused on hospitalisations for two kinds of cardiovascular disease, one of the most prevalent disease groups, over a 20-year period (1992–2011 inclusive). Within this analysis we explore age-stratified response variation due to Indigenous status and sex. The study was approved by the Northern Territory’s Top End Human Research Ethics Committee (approval number HREC12/1750).

## 2. Methods

### 2.1. Admissions Data

**Figure 1:**
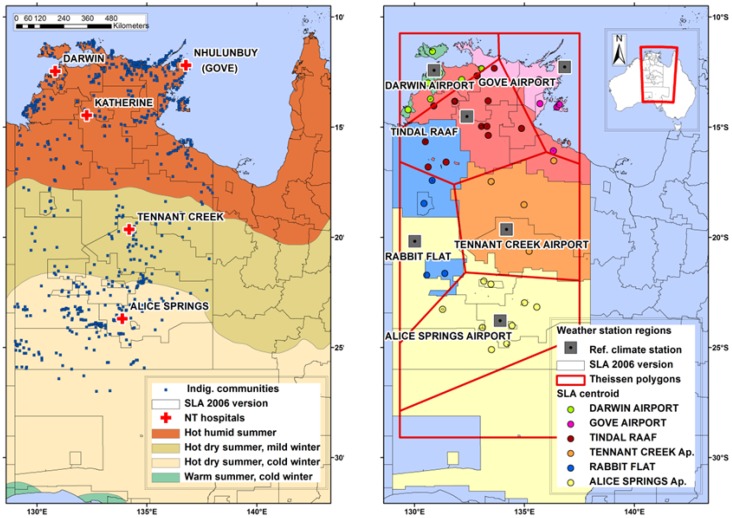
(**Left**) Location of hospitals from which admissions data were accessed denoted by red crosses, and position of Indigenous communities in blue squares overlaying the temperature and humidity zone map of Australia [41] and SLA boundaries (2006) denoted by black lines. (**Right**) Climate reference weather stations denoted by grey squares on which Thiessen polygons denoted by red lines are based. SLA centroids denoted by coloured circles which informed the alignment of each SLA into a polygon region.

Our data cover all admissions from five hospitals in the Northern Territory—Royal Darwin, Gove, Katherine, Tennant Creek and Alice Springs—for the period 1992–2011 inclusive (Figure 1, left hand side). The data included the categories of: date of admission, patient age, sex, Indigenous status, and admissions diagnosis codes. 

Boundaries for the 132 Statistical Local Areas (SLA) were identified for the census year 2006 in the admissions time-series [39]. SLAs are the geographic regions defined for census assessments by the Australian Bureau of Statistics [40] and these were included in the admissions dataset to describe the patient’s place of residence (Figure 1). Admissions data were restricted to patients with residential status in the Northern Territory SLA and Northern South Australia (these included the two SLAs where patients were admitted to Alice Springs hospital for secondary care). 

### 2.2. Diagnosis Codes: Hospital Admissions

Diagnoses codes within the admissions data were identified by the International Classification of Diseases (ICD) [42]. Data collected prior to 30 June 1998 used the 9th ICD Revision ICD-9 codes, and data collected after 1 July 1998 used the 10th ICD-10 codes.

We included in the analyses the cardiovascular diseases IHD (I20–I25, 410–414) and heart failure (I50, 428) because they satisfied the following criteria:

- existing epidemiological evidence for a morbidity association with ambient temperature;
- climatic conditions are likely to affect the physiological function of people with these medical conditions;
- the medical condition is common or significant in a Northern Territory health context; and/or
- the medical condition is prevalent within the Indigenous population.

The IHD codes describe angina pectoris, acute myocardial infarction and other acute cardiac ischaemic events and complications. Heart failure describes all types of congestive and unilateral heart failure. Cardiac arrest (ICD 427.5/I46) was considered but not included because only 92 cases occurred in the 20 years and the ICD-9 and 10 codes do not clearly map to one another.

### 2.3. Population

Time series population profiles by age, sex and Indigenous status for the Northern Territory and South Australia for the census years 1991, 1996, 2001, 2006 and 2011 were obtained from the Australian Bureau of Statistics. These data were categorised using the 2006 SLA boundaries [43]. A linear interpolation of populations was calculated for the intervening years between census dates. Annual population estimates were matched to the admissions data for the relevant region, year, Indigenous status and sex. Population data were used along with the admissions data to determine the rate of admissions per 100,000 people per day.

### 2.4. Geographic Extent of the Study and Climate Data

Three broad climate classifications exist in the Northern Territory and northern South Australia: a hot-humid summer climate in the north, a hot-dry summer, mild winter climate through the middle of the Territory, and a hot-dry summer, cold winter climate in the south [44] (Figure 1, left panel). 

Daily weather records were obtained from the Australian Reference Climate Station (RCS) Network which provides high quality, long-term climate records [44]. Climate records for the Northern Territory and northern South Australia were available from eight weather stations from the RCS (Figure 1, right panel). From north to south, these are: Darwin Airport, Gove Airport, Tindal RAAF, Tennant Creek Airport, Rabbit Flat, and Alice Springs Airport.

An estimate of exposure to daily temperature variation for people living in communities, out-stations and towns across the whole of the Northern Territory was required for this assessment. Therefore, daily maximum temperature (T_max_, °C) and daily minimum temperature (T_min_, °C) were obtained for each weather station. T_max_ was calculated from records taken in the 24 hours from 9 a.m., and T_min_ was calculated from records taken in the 24 hours to 9 a.m. records. For each weather station, the 5th percentile “very cold” and the 95th percentile “very hot” were calculated for all variables. 

Percentiles were used rather than absolute thresholds to account for acclimatisation within the region, after Vaneckova and Bambrick [18] and because, in the absence of a universal definition for a heatwave or temperature extreme, percentile thresholds are better at defining extremes than absolute thresholds when comparing across different climatic zones [45].

The motivation for this study was to determine whether changes in climate would affect future patterns of morbidity. Therefore the effects of temperature parameters alone, and not humidity related variables, were assessed as projections for changes to humidity for this region indicate little change in the future [29].

### 2.5. Spatial Analysis

The location of the weather stations, the most limiting of the two datasets, informed the regional break-up for determination of the population’s exposure to temperature and humidity conditions. Thiessen polygons [46] were employed to divide the Northern Territory into regions, where each polygon contained only one weather station, with any location within a polygon closer to its associated station than to the station in any other polygon (Figure 1, right panel). Since climate observations needed to relate to the population and admissions data in the SLA, centroids of each SLA were calculated and aligned with the relevant weather station regions defined by the polygons. Subsequently, informed adjustments to the alignment were made for nine of the study SLA, to ensure their major population loci sat within the climate zone best reflected by the RCS weather station data. 

Climatic “extremes” should be considered region by region due to an expected level of acclimatisation in the resident population [47,48], therefore “extreme” percentiles were calculated relative to each climatic region.

### 2.6. Data Analysis

Admission rates were calculated by dividing the frequency of admissions per day by the average population, estimated for each year. Data, grouped by Indigenous status and sex, were presented as admission rates per day per 100,000 of the respective population. The data were age-stratified given the anticipated differential response. Those who were under 25 years old were excluded due to too few cardiovascular events, while the remaining admissions, those aged 25 to 64, considered the younger cohort, and those 65 years and above, considered the older cohort, were analysed separately.

To assess climate related effects on health it was assumed that the hospital patient was exposed to the climate of the SLA region of residence immediately prior to their admission to the hospital. Daily admissions from the weather station regions were extracted from the admissions dataset using the selected ICD codes and merged with the respective weather information. In this way, each admission was identified as being made on an “extreme”: either very hot or very cold day, the 5th or 95th percentile; or a “non-extreme”, the remaining central 90 percentile of data.

We used a Poisson loglinear model to calculate the ratio of rates of admissions in each temperature category (rate ratio). The rate for the “very hot” and “very cold” periods divided by the rates for the “non-extreme” periods indicated the responses to climate variation, with numbers greater than one associated with increased rates, and numbers less than one, decreased rates. Rate ratios are presented for different age cohorts and Indigenous status for hospital admissions on extreme compared with non-extreme days. 

The dependent variable was the count of hospital admissions and the independent variables included the indicator of the extreme day, as a categorical variable, defined for each climate variable. Rate ratios were calculated using Chi-square statistics and employed confidence interval levels of 95%. Significance of different frequency of admissions was also tested using an independent samples t-test and assuming unequal variances. Since the Poisson estimates are calculated separately at each point, we accept that we may lose some of the “shared information” we could obtain from a two stage hierarchical model for example [49,50] and this could potentially bias our estimates. 

A formal test to determine whether the data were over-dispersed was carried out by performing a likelihood ratio test between a “standard” Poisson regression and a negative binomial regression. As no improvement was detected by using the latter test, over-dispersion was ruled out.

Trends in admissions data were accounted for by assessing the admissions rate (admission/100,000 population). Over the period of the study a negligible cooling trend in T_max_ was measured (0.1 °C per decade) and in T_min_ (0.2 °C per decade) on average over the Northern Territory region.

Data were extracted from the admissions dataset (~1.5 million admissions) using R software [51]. Statistical analyses were undertaken using SPSS software [52] and spatial analysis undertaken with ArcGIS software [53].

## 3. Results

### 3.1. Population

The average population total in the Northern Territory over the study period was 176,000, with about 42% of the population self-identifying as Indigenous [54]. Population characteristics vary for each region (Table 1), with the Darwin Airport region having the largest population, and by far the highest proportion of non-Indigenous people. The Alice Springs region has the largest Indigenous population. The Gove, Tennant Creek and Rabbit Flat regions have a high Indigenous to non-Indigenous population ratio, but lower numbers overall. The region defined by the Rabbit Flat weather station has a very small population and very few non-Indigenous people, with an average population over this period of about 2,700.

### 3.2. Climate

**Figure 2:**
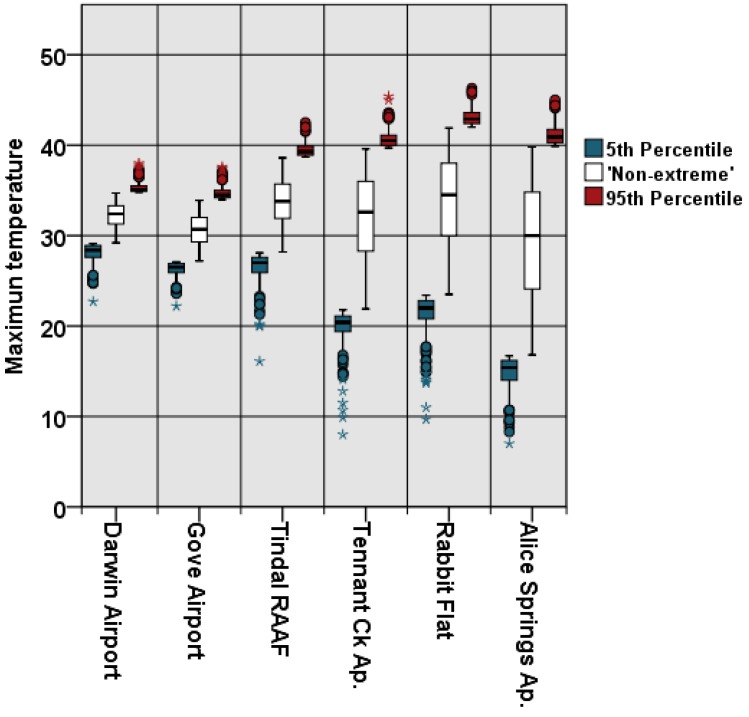
Range of T_max_ (°C) from the northern-most region (left), to the southern-most region (right). Divisions of data into percentile groupings varied accordingly.

Maximum temperatures were highest for the Rabbit Flat region between November and January; with minimum temperatures lowest in the Alice Springs region in July. Extreme percentiles vary significantly across the study region where; for example; the 95th percentile for maximum temperature varies from 34.8 to 38.0 °C in the Darwin region; to 42.0 to 46.3 °C in the Rabbit Flat region (Figure 2). 

### 3.3. Admissions: Indigenous Status, Age and Sex

From a total of almost 1.5 million hospital admissions, about 16,000 for the period 1992–2011 were recorded for cardiovascular diseases, with the majority diagnosed with IHD (Table 2). Absolute admission numbers are higher for males. More IHD admissions occurred in the non-Indigenous population, and more admissions for heart failure in the Indigenous population. 

**Table 1:** Indigenous and non-Indigenous populations in each weather station region and age category averaged over the time period of the admissions records.

Average Population 1992–2011	Row Labels	0 to 24	25 to 44	45 to 64	65 plus	Total
Darwin Airport	Indig.	6,821	3,530	1,550	282	12,183
	non-Indig.	30,697	30,746	19,093	4,034	84,570
**Darwin Airport Total**		**37,518**	**34,276**	**20,643**	**4,316**	**96,752**
Gove Airport	Indig.	6,976	3,657	1,408	292	12,332
	non-Indig.	2,018	2,305	1,297	80	5,700
**Gove Airport Total**		**8,994**	**5,962**	**2,705**	**372**	**18,032**
Tindal RAAF	Indig.	4,381	2,076	834	210	7,501
	non-Indig.	3,196	3,367	1,898	395	8,856
**Tindal RAAF**		**7,577**	**5,444**	**2,732**	**604**	**16,356**
Tennant Ck. Airport	Indig.	1,954	973	420	124	3,469
	non-Indig.	695	708	528	98	2,028
**Tennant Ck. Airport**		**2,648**	**1,681**	**947**	**222**	**5,498**
Rabbit Flat	Indig.	1,191	541	252	87	2,070
	non-Indig.	206	219	147	19	591
**Rabbit Flat Total**		**1,397**	**759**	**399**	**106**	**2,662**
Alice Springs Airport	Indig.	7,900	4,334	1,844	585	14,664
	non-Indig.	8,050	8,530	5,283	1,015	22,878
**Alice Springs Airport Total**		**15,950**	**12,864**	**7,127**	**1,600**	**37,541**
Indig. Total		29,222	15,111	6,307	1,578	52,219
Non-Indig. Total		44,862	45,875	28,245	5,641	124,623
**Overall Total**		**74,084**	**60,986**	**34,552**	**7,219**	**176,841**

**Table 2:** Number of admissions for each diagnosis grouping for 1992–2011, all regions.

Diagnosis	Age Group	Indigenous			Non-Indigenous		
		Female	Male	Total	Female	Male	Total
Heart failure N = 3,847	25–64	809	1,284	2,093	83	326	409
	65 plus	216	149	365	377	603	980
	**Total**	**1,025**	**1,433**	**2,458**	**460**	**929**	**1,389**
Ischaemic heart disease N = 12,026	25–64	1,907	2,472	4,379	992	3,228	4,220
	65 plus	329	324	653	953	1,821	2,774
	**Total**	**2,236**	**2,796**	**5,032**	**1,945**	**5,049**	**6,994**

**Figure 3:**
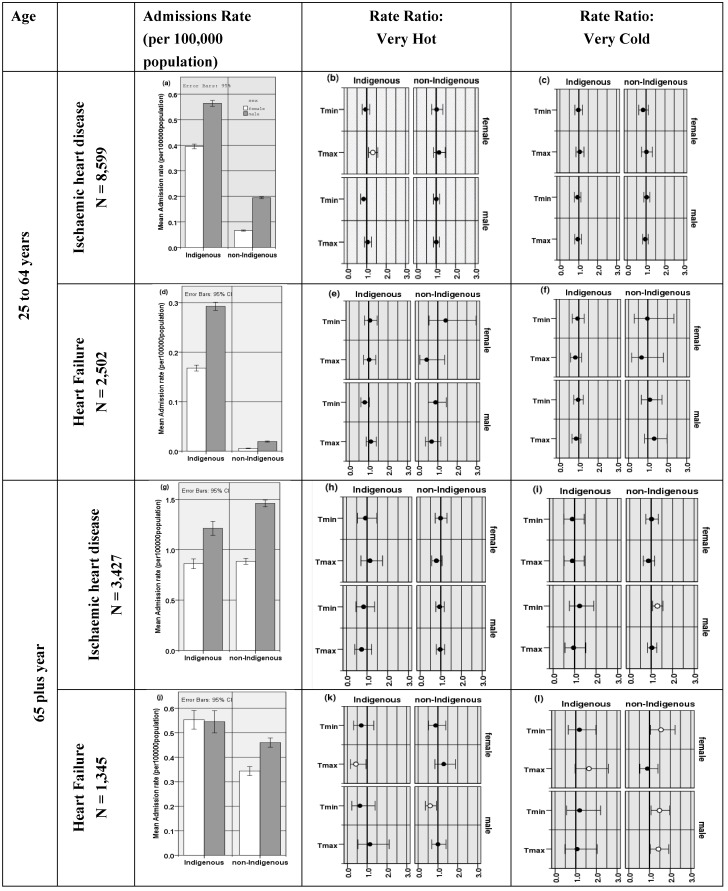
Northern Territory hospitals IHD and heart failure admission rates for 25 to 64 years (upper) and 65 plus years (lower). Female (white) and male (grey) are shown. Note that the scales vary in the left panel. Female and male, Indigenous and non-Indigenous Rate ratio plots for T_max_ and T_min_ are shown for very hot (mid panel) and very cold days (right panel) with significant effects depicted by an open circle. 95% confidence intervals are shown.

Rates of admission for both younger and older cohorts are shown for IHD and heart failure in Figure 3 (left column). Rates of admission for heart failure were much lower than for IHD, and also lower in the younger age cohort. Over all cohorts, IHD admission rates were highest in older non-Indigenous males (Figure 3g), while heart failure rates were higher in the older Indigenous population (Figure 3j). 

In the younger cohort, Indigenous admission rates were much higher than non-Indigenous, and males greater than females (Figure 3a,d). Admission rates increase in the older cohort (65 plus) with less difference between the Indigenous compared to the non-Indigenous population than in the younger cohort (Figure 3g,j). Notably, relative rates of female admissions increase compared to males for heart failure as the population ages. 

### 3.4. Sensitivity of Admission rate to Climatic Variation

#### 3.4.1. Younger Cohort

In the younger cohort, a 17% higher rate of IHD admissions was found with high maximum temperatures if both sexes were considered (*p* = 0.015, not shown), with a significant increase of 32% for the Indigenous females cohort (95% CI 10–56, *p* = 0.002) (Figure 3b). Indigenous males also showed a tendency for a higher rate of admissions on hotter days, although this result was not significant. 

For heart failure, a non-significant tendency for fewer admissions during very hot conditions in the non-Indigenous younger cohort contrasted with a tendency for more admissions in the Indigenous cohort. 

In the younger cohort, while there was a non-significant increased rate of admissions for heart failure in the non-Indigenous male population in cold conditions (Figure 3f), no other group was affected.

#### 3.4.2. Older Cohort

The pattern of response to temperature was different in the older age cohort (65 plus) compared to the younger for both IHD and heart failure. Admissions for IHD for non-Indigenous men showed increased rates of 29% (95% CI 6%–55%, *p* = 0.008) were associated with low T_min_ (Figure 3i). For Indigenous and non-Indigenous females, no temperature response was noted for IHD admissions (Figure 3h,i). 

Heart failure admissions in the older female cohort were reduced by 63% for Indigenous females in hot weather (95% CI 14%–89%, *p* = 0.047) (Figure 3k) and increased for Indigenous females in cold conditions by 64 % (95% CI −2%–158%, *p* = 0.045) (Figure 3l). Non-Indigenous females showed increased admissions on very cold days (56% increase, 95% CI 4%–123%, *p* = 0.02) (Figure 3l).

For heart failure in males, non-Indigenous older males have 40% lower admission rates associated with higher T_min_ (95% CI 7%–63%, *p* = 0.03) (Figure 3k), with increased admissions on very cold days (Figure 3l).

## 4. Discussion

To our knowledge this is the first quantitative study undertaken that investigates how ambient temperature affects the health, specifically IHD and heart failure, of disaggregated Indigenous and non-Indigenous populations. Estimates of temperature-morbidity associations suggest how projected climate change could affect future patterns of morbidity, and particularly whether concerns of a disproportionately greater effect on Australia’s Indigenous population living in the Northern Territory, is well-founded. 

### 4.1. Admission Rates: Indigenous vs. Non-Indigenous

The higher rates of admissions for IHD among males, and higher rates for older ages reported here, are consistent with previous studies [55,56]. Higher Indigenous admission rates compared to non-Indigenous rates are not surprising as the incidence of IHD is associated with diabetes, the prevalence of which is three times higher in the Indigenous population [57]. Incidence of this disease is also associated with poor diet, again a significant factor in the Indigenous population [58]. 

Previous studies have also reported the disparity between Indigenous and non-Indigenous admission rates [24,59] with hospitalisations for IHD being more than twice as high for Indigenous compared to non-Indigenous males, and four times as high for Indigenous females [57]. Interestingly, however, for the 65 plus cohort, admission rates for IHD were higher among the non-Indigenous population. This may be because relatively few Indigenous Australians survive past 65 years when hospitalisation for IHD is more likely [60]. Over the period of this study, for instance, we found that while more than half of the youngest cohort (0 to 24) were Indigenous, only about a quarter of the older age cohort, those above 65 years old, were Indigenous, indicating a large difference in survival rates and life expectancy. The finding of a cold effect among older people in this study is consistent with the literature, but the low numbers in this age group mean there is less confidence in these results.

In the Northern Territory, the rate of admissions for heart failure was notably higher in the younger Indigenous population cohort, and slightly higher in the older Indigenous population cohort, compared to the non-Indigenous cohort. Again the difference in Indigenous *vs.* non-Indigenous rates has been reported previously [24]. The increased rate of admission for heart failure we found may be attributable to lifestyle and socioeconomic factors [61]; for instance, in 2004–2005 half of Indigenous adults were regular smokers—twice the rate of non-Indigenous adults [57]. In addition, slightly more than half of Indigenous people aged 15 years and over were overweight or obese. And while the rates of obesity for Indigenous and non-Indigenous men were similar, Indigenous women were around one-and-a-half times as likely as non-Indigenous women to be overweight or obese [57]. 

Finally, while a much higher rate of younger males were admitted with heart failure, consistent with Aronow *et al.* [62], in the older population the rates for females had increased relative to males, and were the same in the older Indigenous population. Women tend to survive longer after the onset of heart failure and tend to be older when first diagnosed [63], which would account for this result. 

### 4.2. Admission Rates: Sensitivity to Ambient Temperature

In Australia, it is understood that health effects of heat are influenced by variation in regional tolerances [31], by season [14,21], and a person’s age, sex and socioeconomic factors [17,64]. Comparing climatic effects on health outcomes between the Indigenous and non-Indigenous population contributes to understanding these effects in an Australian context. 

In the 25 to 64 year old cohort, a significant 17% increase in admission rates for IHD was detected in the Indigenous population on very hot days. Other studies have also reported heat associated increased admissions for IHD in other populations [65]. Here, Indigenous females (32% increase, *p* < 0.05) were most affected by higher maximum temperatures. Several other studies also found an increased female vulnerability to heat [2] that could be explained by reported higher rates of overweight females compared to males in the Indigenous population [57]. A recent study of “all cause” hospital admissions among a smaller group of Indigenous people [32] in the Northern Territory found men were more susceptible to heat than women. 

We propose ambient heat would not be a direct cause of IHD, but would increase physiological stress, with other pre-existing health issues potentially compounding the effects of heat. High temperatures cause peripheral vasodilatation, an increase in cardiac output and sweating to maintain temperature homeostasis [66] which, perhaps associated with dehydration, increase cardiac work load in an already diseased heart.

In the 65 plus age cohort we found a contrasting response to climatic conditions compared with the younger cohort. In general, a tendency for an increased rate of admissions for the cardiovascular diseases studied was observed in response to cold conditions, with a reduction in admissions on very hot days. The most notable increase was found in the Indigenous and non-Indigenous older male populations. This tendency was also apparent for females being admitted for heart failure. This finding is consistent with other studies showing the positive association of cold temperatures on rates of admission for heart conditions [21,67]. While it is surprising that conditions may get cool enough in the Northern Territory to cause these adverse effects, it has been found that paradoxically, excess winter mortality from cardiovascular disease is actually more pronounced in countries having mild rather than cold winters [68] and in warmer regions of Austalia [31]. 

In cold conditions the primary autonomic defences are vasoconstriction and shivering [69]. The cold sensitivity response derives from a combination of cardio-respiratory response, blood clotting, renal and immune effects. These increase heart rate and blood pressure while reducing cardiac muscle contractility, deplete blood volume, increase blood viscosity and, if core temperature falls, induce immunological changes [70,71]. Cold conditions may have a stronger influence on increasing morbidity associated with heart failure, compared to IHD, because of the extra effort required for the heart to function under these conditions. It is interesting to note that in the older population, on hotter days there is a tendency towards lower admissions for heart failure. 

As the Indigenous population comprises almost a third of the population of the Northern Territory [72], it was an ideal region to study the effects of ambient temperature on hospital admissions for this kind of disaggregated population assessment. Conducting an epidemiological analysis over a region with such a diverse range of climate types required careful consideration of the methodological approach taken. Dividing the region into geographic areas defined by both population distribution and climate zone, whereby the most relevant weather data were applied to the population being admitted to hospital, accommodates the well understood concept that populations have a degree of acclimatisation to their usual region of residence [31], at the same time as measuring exposure as accurately as possible. This methodology also enabled avoidance of any averaging of the daily time-series weather records. This means that weather that is “extreme” by a local standard was captured. 

As the population density of some of the regions was very low, it was not possible to assess the response at a regional level, so results were summarised and presented for the whole study area. We acknowledge that the response may, however, vary from region to region, and this could be an area of further study. 

There are a number of limitations to this study due to data constraints. While sex and age were included in the analysis, information was unavailable on other factors affecting cooling capacity or response, such as obesity, medication, fitness and pregnancy as well as a range of lifestyle and socio-economic factors that were out of the scope of this analysis [7,64,73]. We were not able to include morbidity occurring outside hospital, and hospital admissions tend to reflect only the more severe episodes of illness. The diagnosis recorded in the admissions data may not be the condition for which admission actually occurred and, while unable to assess this, we assumed it was related to the reason for admission. Mortality data were also not available for this study and this would potentially contribute to our understanding of the effects of temperature on Indigenous health.

The relatively small population of the Northern Territory meant that some additional and potentially enlightening analyses on regional effects were not undertaken. Small populations also dictated the need to create larger age bands to conduct meaningful analyses rather than more refined age group analyses such as splitting younger and middle-aged groups and including a “very old” over 85 years group. Furthermore, the low population density meant that very large areas with limited climatic monitoring were studied. The lack of topographic relief through the region, resulting in a more uniform climatic signal, does mean that the climate data used were a relatively good fit across the region. 

Finally, limitations exist relating to the potential confounding effects of air pollution, primarily particulates but also gases, for example ozone. While we recognise the relationship between particulate pollution from bush fires and heart disease [74], and that there might be a relationship to cooking fires, beyond crude seasonal interferences such data were unavailable.

## 5. Conclusion

Overall, we found rates of hospitalisation to be higher for older people compared to younger people, Indigenous people compared to non-Indigenous people, and males compared to females. In general, the younger (25 to 64 year old) Indigenous population tended to have higher rates of admissions on very hot days, and the older (65 plus) non-Indigenous male population had higher rates of admission on very cold days. These findings support widespread concerns over the likely disproportionate health impacts on Indigenous people from climate change, in particular for the cardiovascular conditions studied here.

Climate change is projected to increase the frequency of hot days, and decrease the frequency of cold days in this region. These projections suggest that there may be an increase in admissions for IHD in younger Indigenous people but reduced admissions in older populations for the heart conditions studied here. The increasing temperature extremes projected by climate models to occur within the lifetime of teenagers today suggests that further examination of these effects is warranted.

In the absence of mitigation and adaptation activities, climate change is thus likely to have greater adverse health effects—at least for cardiovascular related events – in the Indigenous compared to the non-Indigenous population in the Northern Territory, with the increase in heat related admissions in the younger population likely to outweigh any reductions in admissions among older Indigenous people, especially from a burden of disease perspective. 
